# Chemical Clearing and Dehydration of GFP Expressing Mouse Brains

**DOI:** 10.1371/journal.pone.0033916

**Published:** 2012-03-30

**Authors:** Klaus Becker, Nina Jährling, Saiedeh Saghafi, Reto Weiler, Hans-Ulrich Dodt

**Affiliations:** 1 Vienna University of Technology, FKE, Department of Bioelectronics, Vienna, Austria; 2 Center for Brain Research, Medical University of Vienna, Vienna, Austria; 3 University of Oldenburg, Department of Neurobiology, Oldenburg, Germany; Emory University, United States of America

## Abstract

Generally, chemical tissue clearing is performed by a solution consisting of two parts benzyl benzoate and one part benzyl alcohol. However, prolonged exposure to this mixture markedly reduces the fluorescence of GFP expressing specimens, so that one has to compromise between clearing quality and fluorescence preservation. This can be a severe drawback when working with specimens exhibiting low GFP expression rates. Thus, we screened for a substitute and found that dibenzyl ether (phenylmethoxymethylbenzene, CAS 103-50-4) can be applied as a more GFP-friendly clearing medium. Clearing with dibenzyl ether provides improved tissue transparency and strikingly improved fluorescence intensity in GFP expressing mouse brains and other samples as mouse spinal cords, or embryos. Chemical clearing, staining, and embedding of biological samples mostly requires careful foregoing tissue dehydration. The commonly applied tissue dehydration medium is ethanol, which also can markedly impair GFP fluorescence. Screening for a substitute also for ethanol we found that tetrahydrofuran (CAS 109-99-9) is a more GFP-friendly dehydration medium than ethanol, providing better tissue transparency obtained by successive clearing. Combined, tetrahydrofuran and dibenzyl ether allow dehydration and chemical clearing of even delicate samples for UM, confocal microscopy, and other microscopy techniques.

## Introduction

In the recent years, GFP has become a common reporter gene in molecular biology, medicine, and cell biology [1]. Not least, this was due to the generation of GFP expressing transgenic mouse lines, allowing histological studies of neuronal networks with unmatched precision in vivo and in vitro [2]. However, GFP is quite unstable with respect to some chemicals routinely applied in histology. This is a severe drawback, since many histological techniques, such as tissue clearing, staining, and paraffin or resin embedding depend on careful foregoing tissue dehydration, and thus often fail when applied to GFP expressing samples [3]. Particularly ethanol, being the most common tissue dehydration medium, can cause a markedly decline, or even a complete destruction of GFP fluorescence, if not applied very carefully.

Light microscopy techniques, as confocal microscopy [4] or ultramicroscopy (UM) [5], [6] require sufficiently transparent specimens. Since most biological specimens are opaque, chemical clearing is a widespread technique to compass this problem. The effect of chemical clearing relies on immerging samples in a medium having a refractive index similar to proteins. This way, light scattering is considerably reduced, so that the specimens become transparent, if light absorption by chromophores is moderate. The commonly applied clearing medium consists of one part benzyl alcohol and two parts benzyl benzoate (BABB). BABB is unmixable with water and thus can only be applied after careful tissue dehydration. As ethanol, exposure to BABB can cause a noticeable decline, or even a complete vanishing of GFP fluorescence, if the incubation times are chosen too long, or if the GFP expression rate in the specimen is low. Thus, we screened various chemicals to find more GFP friendly substitutes for BABB-clearing and ethanol-dehydration.

## Results

### GFP-friendly tissue dehydration with tetrahydrofuran (THF)

**Figure 1:**
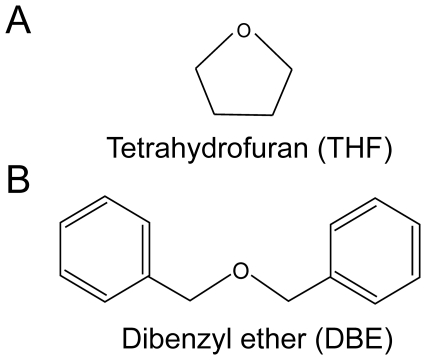
Chemical structures of tetrahydrofuran (THF) and dibenzyl ether (DBE). **A**: THF. **B**: DBE. Interestingly, both compounds are ethers without further functional groups.

THF is a colorless, flammable, cyclic ether with an ethyl ether like odor (**Figure 1**). It is mixable with water in any proportions, and thus can be applied, as ethanol, to stepwise displace the water in biological samples. The use of THF for tissue dehydration has rarely been suggested before as a substitute for the highly toxic paraffin embedding intermedium dioxane [7], [8], [9]. However, it never found a widespread application in histology, and to our knowledge, has never been applied for GFP-friendly specimen clearing before.

To assess the compatibility of GFP with THF dehydration, the left hemispheres of two GFP-expressing paraformaldehyde fixed mouse brains (thy-1 EGFP-M c57/bl6 mice [10]) were dehydrated in graded series of peroxide-free THF, while the corresponding right hemispheres were dehydrated in graded series of ethanol as a control. As a further control, we dehydrated both hemispheres of two further mouse brains with ethanol.

**Figure 2:**
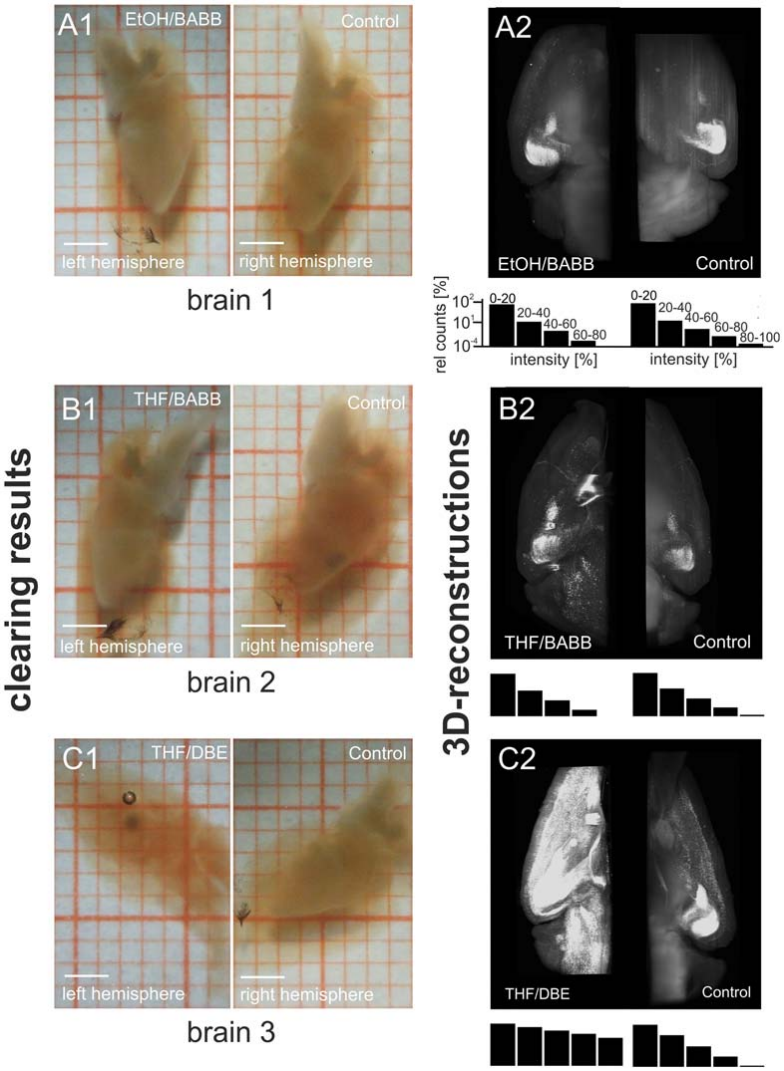
Comparison of THF/DBE clearing with ethanol/BABB. **A–B**: Left and right brain hemispheres from two mice (postnatal day 101). The left hemispheres of each animal were dehydrated with ethanol, or THF. The corresponding right hemispheres were dehydrated with ethanol as a control. Afterwards, the hemispheres were rendered transparent with BABB (A1 and B1. **C**: Left and right brain hemisphere of an exemplary mouse (postnatal day 101). The left hemisphere was dehydrated with THF and cleared with DBE. The right hemisphere was dehydrated with ethanol and then cleared with BABB as a control (C1). From all hemispheres 3D reconstruction were performed by UM (A2–C2, Olympus objective XLFluar 4×, N.A. 0.28). As evident from A–C, THF-dehydration provides an improved fluorescence signal (B2), and considerably reduced background fluorescence (B2), while DBE-clearing provides considerably better tissue transparency (C1) and strikingly better fluorescence preservation (C2). The black bars below A2–C2 represent intensity histograms of the respective image stacks used for 3D reconstruction. Length of scale bars in Figures A1–C1 is 2 mm. Scales are identical for all histograms A2–C2 and thus are only shown in the upper two histograms.

After dehydration, the brain hemispheres were rendered transparent with BABB, and 3D UM-reconstructions were performed as described in [5]. The tissue transparency obtained by BABB clearing was approximately the same for the ethanol dehydrated and the THF dehydrated hemispheres (**Figure 2 A1–B1**). This indicates that the dehydration by THF was similarly successful than dehydration by ethanol. Otherwise, possibly existing residuals of water would have caused a noticeable tissue-diffusion after clearing. However, 3D UM-reconstructions of the THF dehydrated hemispheres showed considerably reduced background fluorescence intensity, and markedly improved fluorescence signal intensity compared to those hemispheres dehydrated by ethanol (**Figure 2 A2–B2**).

### GFP friendly tissue clearing with dibenzyl ether

Phenylmethoxymethylbenzene (dibenzyl ether, DBE, CAS 103-50-4) (Sigma-Aldrich Austria, Order-No. 108014) is a colorless cyclic ether (**Figure 1B**). Its refractive index (*n* = 1.562) is slightly higher than the refractive index of BABB (*n* = 1.559).

To assess the viability of DBE as a tissue-clearing medium, we dehydrated the hemispheres of two mouse brains (thy-1 EGFP-M (c57/bl6) mice) with THF. The two left hemispheres were cleared with peroxide free DBE and the corresponding right hemispheres were cleared with peroxide free BABB as a control. As illustrated by **Figure 2 B1–C1**, the hemispheres cleared with DBE became more transparent than the hemispheres cleared with BABB. After clearing, 3D UM-reconstructions of the brain hemispheres were performed [5]. Visual inspection exhibited a much brighter and more extended fluorescence signal in the hemispheres rendered transparent with DBE (**Figure 2 B2–C2**).

**Figure 3:**
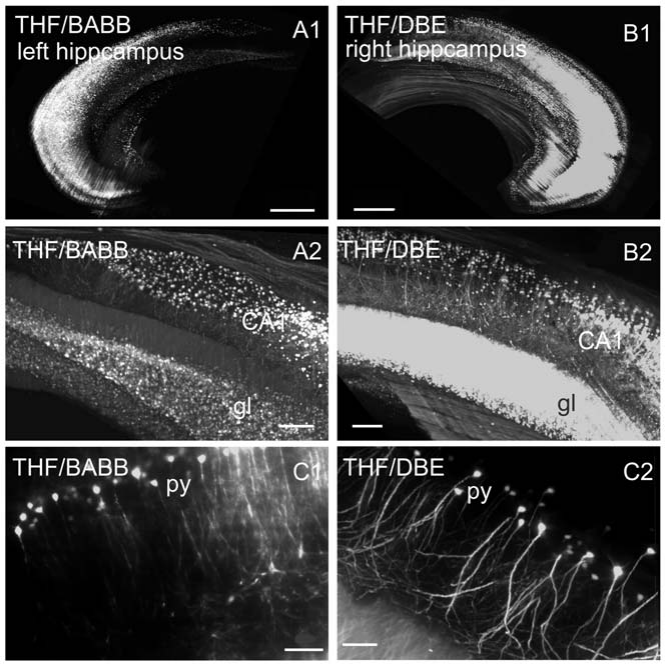
UM-reconstructions of isolated hippocampi of a thy-1 EGFP-M mouse. DBE clearing provides improved fluorescence and visibility of details. **A**: left hippocampus dehydrated with THF and cleared with BABB. **B**: corresponding right hippocampus dehydrated with THF and cleared with DBE. **A1–B1**: Olympus objective XLFluar 4×, N.A. 0.28, scale bar 100 µm. (**A2–B2**) 4× Olympus objective XLFluar 4×, N.A. 0.28, scale bar 20 µm. **A3–B3**: 4× Olympus objective XLFluar 4×, N.A. 0.28, scale bar 10 µm. **CA1**: cornu ammonis region one. **gl**: granular cell layer **py**: pyramidal cells.

We further prepared isolated mouse hippocampi [6], dehydrated them with THF, and cleared them with BABB (left hippocampus), or DBE (corresponding right hippocampus). As in the brain hemispheres, UM-reconstructions [6] verify the strikingly improved fluorescence signal intensity obtained by DBE-clearing (**Figure 3**).

### Further chemicals tested

In order to find a more GFP-friendly tissue dehydration medium, we further checked methanol, acetone, 2-butoxyethanol, dimethylformamide, dimethylsulfoxide (DMSO), and dioxane. For GFP-friendly chemical clearing, we further tested methylsalicylate (*n* = 1.518), benzyl alcohol (*n* = 1.540), benzyl benzoate (*n* = 1.569), trans-anethole (*n* = 1.561), isoeugenol (*n* = 1.578), isosafrol (*n* = 1.573), 1,5-dibromopentane (*n* = 1.513), and bromobenzene (*n* = 1.560) (all chemicals obtained from Sigma-Aldrich, Austria). As in the experiments before, the left hemispheres of mice brains were treated with the respective test substance. The corresponding right hemispheres were treated with ethanol/BABB as a control.

**Figure 4:**
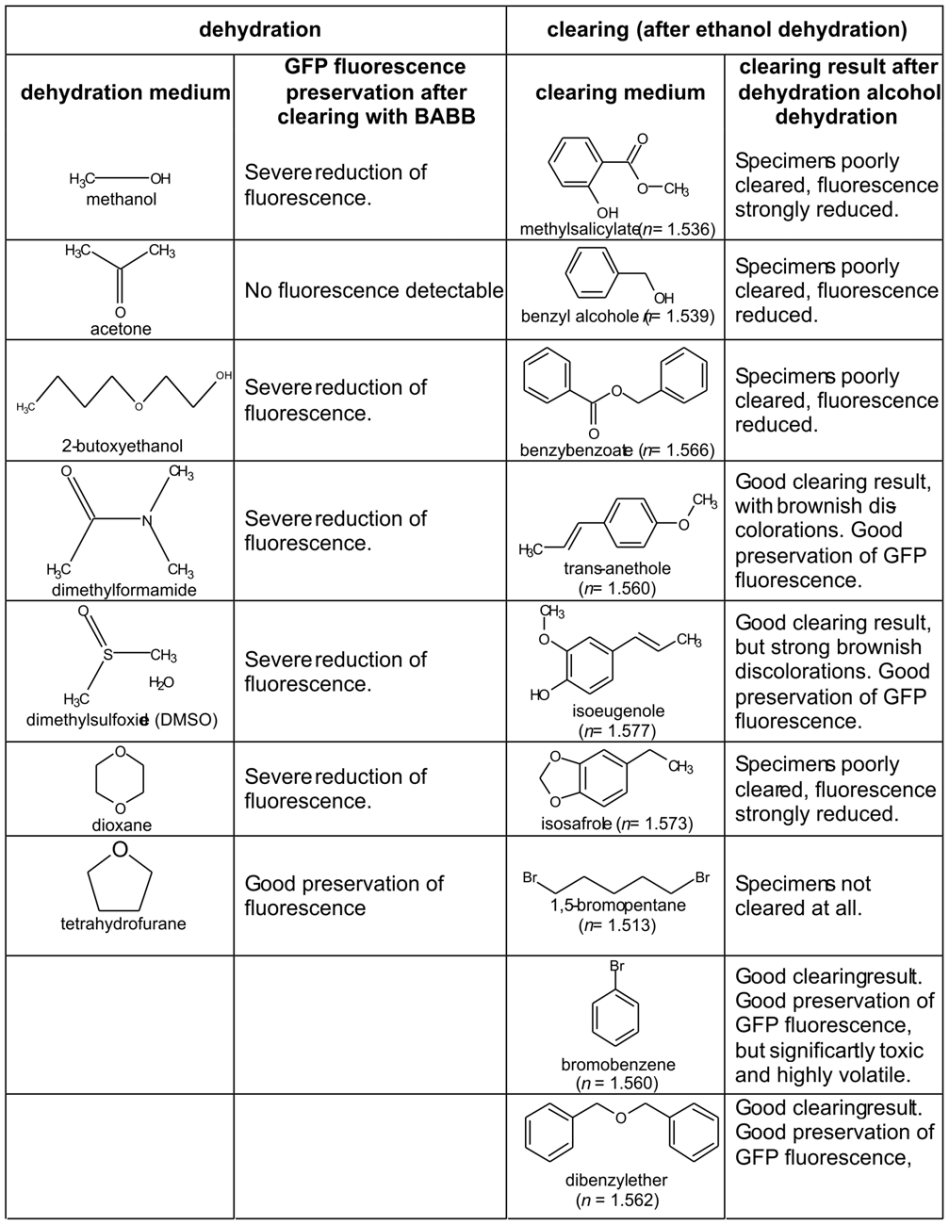
Dehydration and clearing chemicals tested. Most of the substances found to be GFP-incompatible possess hydroxyl, or carbonyl groups.

The tested substances for dehydration invariably performed worse than THF. In all cases, GFP fluorescence was either strongly diminished or destroyed (**Figure 4**). However, for clearing, bromobenzene provided results similar to DBE, but due to its significant toxicity and low vapor pressure, we consider it as less suitable than DBE. Trans-anethole and iso-eugenole preserved GFP fluorescence better than BABB, but not as good than DBE. The clearing result obtained with both substances was lowered by strong brownish tissue-discolorations.

## Discussion

In order to find more GFP-friendly dehydration and tissue-clearing media, we tested different chemicals. We found that dehydration with THF provides improved fluorescence intensity and markedly reduced background fluorescence in mouse brains. In a publication in Nature Medicine 2012 about axonal regeneration, authored with our contribution, the advantage of THF as a GFP-friendly tissue dehydration medium was recently demonstrated for the mouse spinal cord [11]. For clearing, DBE provides strikingly improved fluorescence intensity and a noticeably improved tissue transparency.

The tested tissue clearing chemicals were selected from the electronic chemical database Merck Index [12]. Screening was done over about 10000 chemicals, using a refractive index between 1.5 and 1.7 as a selection criterion. The finally tested substances were selected according to the following criteria: a) liquid with preferably low viscosity, b) preferably low toxicity and low volatility, c) affordable price.

The amount of tissue shrinking, being an unavoidable side effect of any clearing procedure involving tissue dehydration, was found to be slightly higher with THF dehydration than with ethanol. However, we found no increase of shrinking artifacts, as tissue disruptions, with THF dehydration. With THF dehydration followed by BABB clearing, the size of dissected spinal cords is reduced isotropically by about 20% in each dimension and about 50% in 3D volume [11]. In contrast to this, the contribution of the clearing medium (BABB or DBE) to the total amount of shrinkage is negligible. The total amount of tissue shrinking essentially relies on the foregoing elimination of water by the dehydration medium.

We found that THF/DBE well preserves fluorescence also in immune-labeled mouse brains [6]. We have not tested nuclear labeling agents as Hoechst or DAPI for their compatibility with THF or DBE up to now.

The clearing process with DBE is faster than with BABB. This may be due to the lower viscosity of DBE and thus an improved tissue penetration. The dynamic viscosities of benzyl alcohol and benzyl benzoate are 6.4 cP and 10 cP, respectively [13]. Since BABB consists of two parts benzyl benzoate and one part benzyl alcohol, its viscosity can be estimated as ∼8,8 cP. The viscosity of DBE is 5.3 cP [13], and thus 40% lower than the viscosity of BABB.

Compared to BABB, DBE is of low toxicity. It has been used in moderate doses as a flavoring agent in food industry since 1940. An animal study found no toxic effects of 196 mg/kg/day DBE in rats [14]. This would correspond to a dose of about 12 g/day in a 60 kg person. The price of DBE is considerably lower than the price of BABB.

THF and DBE are both ethers without further functional groups. Interestingly, most of the substances we found to be GFP-incompatible possesses hydroxyl, or carbonyl groups (**Figure 4**). It may be speculated that the absence of these highly reactive functional groups in THF and DBE contributes to their good GFP-compatibility. Principally, ethers are known to be chemically relatively inert.

Peroxide contaminations in THF, BABB, or DBE should always be removed as described in the methods part, since they can destroy GFP fluorescence even in very low concentrations [15]. Peroxide concentrations can e.g. be measured with Quantofix Peroxide 25 test stripes (Sigma-Aldrich, Austria, Order-No. Z249254).

Other tissue clearing solutions than BABB were developed before. Spalteholz [16], a pioneer in the field of tissue clearing, used methysalicylate and isosafrol, to render anatomical preparations transparent. Both chemicals were shown in this paper to be of poor GFP-compatibility. Recently, a novel water mixable clearing medium based on 4M urea (Sca/e) was suggested [17]. While this mixture may sufficiently clear very young mouse brains below E14 and mouse embryos E13.5, it can already be recognized from Figure 1 in [17] that it does not clear the myelinated regions in 1.5 mm thick brain slices. This is to be expected, as myelin is a fatty substance and thus cannot be cleared without lipophile organic solvents. While we could not yet test it on mouse brains, we looked for the effect of sca/e on muscle preparations. We failed to render cubical muscle tissue samples (∼5–10 mm edge length) transparent by incubation in scale/e for two month. Contra dictionary, control experiments with samples treated with THF/DBE provided excellent clearing results already after a few hours.

We presented a more GFP-friendly dehydration and clearing technique for GFP expressing mouse brains. Beside mouse brains, our technique can also be applied to mouse spinal cords, entire mouse embryos, entire *Drosophila*, and other specimens.

## Materials and Methods

### Preparation of mouse brains

Six mouse brains were obtained from 3–5 weeks old thy-1 EGFP-M (c57/bl6) mice [10]. We killed the mice by CO_2_, and transcardially perfused them post mortem with at least 20 ml 0.1 M PBS (pH 7.6, 1000 Units/ml Heparin) until all blood was removed, followed by 100 ml 4% Paraformaldehyde in 0.1 M PBS for perfusion-fixation. We removed the brains from the skull, and placed them in the fixative for overnight at 4°C, rinsed them 3 times in PBS (30 min each). Animal care and euthanasia was done in accordance with the ethic guidelines of the German Max-Planck society and with the Austrian animal protection law. According to §2 of the Austrian animal experiments act, special approval by an ethics committee or an approval number was not required, since all experiments were performed on isolated organs and not on living animals.

### Dehydration of mouse brains

The fixed brains were split along the median line into their left and right hemispheres. The hemispheres were differently incubated in ascending concentration series of a) ethanol, or b) peroxide free THF (Sigma-Aldrich, Austria Order-No. 186562). The applied concentration steps were 50 Vol%, 70 Vol%, 80 Vol%, 96 Vol%, 3×100 Vol% for ethanol as well as for THF (12 hours per step).

### Clearing of mouse brains

The dehydrated left or right hemispheres were incubated at room temperature in separate glass vials, each containing ∼10 ml peroxide free BABB or peroxide free DBE, respectively. DBE was obtained from Sigma-Aldrich, Austria (Order-No. 108014). The specimens were left in the clearing solution until they became transparent (about 1–2 days). During incubation, the clearing medium was exchanged three times. For better diffusion, the specimen vials were slightly shaken on a vibrating table placed under an extraction hood.

### Preparation of dissected mouse hippocampi

Mouse brains were prepared as described above. The hippocampi were dissected and stored in 4% PFA for post fixation overnight. Afterwards they were rinsed three times in PBS (15 min each) and immediately dehydrated.

### Dehydration of dissected mouse hippocampi

The dissected and fixated hippocampi were incubated in ascending concentration series of peroxide free THF. The applied concentration steps were 50 Vol%, 80 Vol%, 96 Vol%, 100 Vol% THF (1 hour per step, last step overnight).

### Clearing of dissected mouse hippocampi

The dehydrated dissected mouse hippocampi were incubated in BABB or DBE until they became transparent (about 3–6 hours).

### Removal of peroxides

**Figure 5:**
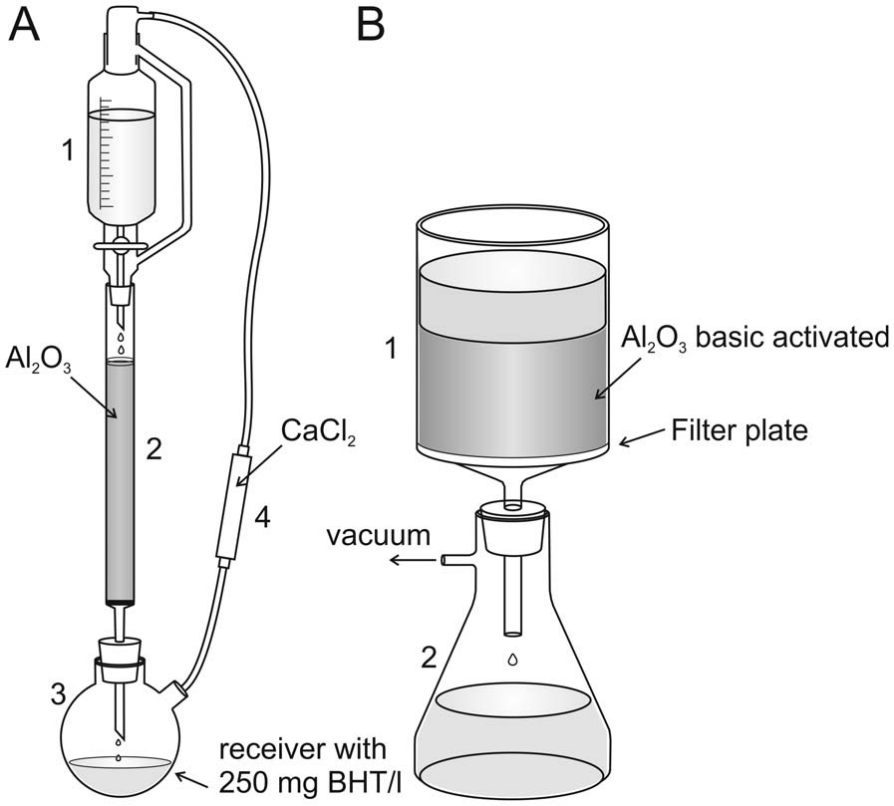
Removal of peroxides from THF and DBE. **A**: Apparatus for peroxide cleaning of THF **1**: Dropping funnel with pressure compensation. **2**: Chromatography column filled with basic activated aluminum oxide activity grade Brockman 1 **3**: Two necked round bottom flask **4**: Drying tube filled with calcium chloride. **B**: Apparatus for peroxide removal in DBE and BABB **1**: Filter unit with filter plate (16–40 µm pore size) **2**: Vacuum tight filtering flask.

Peroxide removal in THF was done by column absorption chromatography (**Figure 5A**) with basic activated aluminum oxide activity grade Brockman I (Sigma-Aldrich, Austria, Order-No. 199443, about 250 g per liter THF) [18]. The stabilizer, which is contained in commercially distributed THF, is also removed during chromatography. This stabilizer is required to prevent the generation of dangerous amounts of peroxides by sunlight or exposure to oxygen. Therefore, it is essential by safety reasons to substitute it, e.g. by adding 250 mg/l butyl hydroxyl toluol (BHT) into the receiver flask (Sigma-Aldrich, Austria, Order-No. W218405). THF, which is insufficiently stabilized can explode after prolonged exposure to oxygen and/or sunlight and may be life dangerous!

Due to their higher viscosity and boiling points, removal of peroxides in BABB and DBE can be done using the setup described in **Figure 5B**. The filter funnel is filled with ∼250 g activated aluminum oxide per liter, and suction is applied to the receiver flask. Peroxide free THF, BABB, or DBE should be stored in brown glass bottles over a molecular sieve with 3 Angstrom mesh wide (Sigma-Aldrich, Austria, 69839) to protect it from water. Contact with oxygen, which would accelerate the reformation of peroxides is prevented by filling the bottles with argon as an inert gas.
