# Correction: Chemical Clearing and Dehydration of GFP Expressing Mouse Brains

**DOI:** 10.1371/annotation/17e5ee57-fd17-40d7-a52c-fb6f86980def

**Published:** 2012-08-07

**Authors:** Klaus Becker, Nina Jährling, Saiedeh Saghafi, Reto Weiler, Hans-Ulrich Dodt

In the Author Contributions section, Saiedeh Saghafi (SS) should be listed as one of the persons who performed the experiments. 

